# Cutaneous bacteria induce immunosuppression

**DOI:** 10.18632/oncotarget.5962

**Published:** 2015-10-04

**Authors:** Yuliya Skabytska, Tilo Biedermann

**Affiliations:** Department of Dermatology and Allergology, TUM School of Medicine, Technische Universität München, Germany

**Keywords:** skin inflammation, cutaneous bacteria, myeloidderived suppressor cells, IL-6, atopic dermatitis

**Figure 1:**
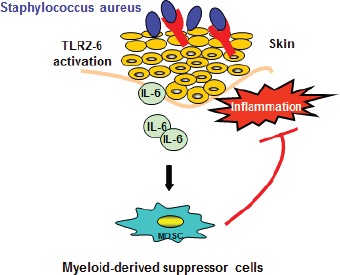
Cutaneous Staphylococcus aureus induce systemic immune suppression Cutaneous exposure to TLR2/TLR6 but not TLR2/TLR1 ligands induces systemic immune suppression following cutaneous bacterial colonization. This is due to a release of IL-6 by keratinocytes, which causes a systemic induction of Gr1^+^CD11b^+^ myeloid-derived suppressor cells (MDSCs) directly suppressing inflammation.

The skin is the largest organ at the interface between the environment and the host with the function of mounting effective host defense. Microbiota and the host immune system are in permanent contact and communication via the skin. The complex relationship between the skin microbiome and cutaneous immunity is poorly understood, however recent evidence supports the idea that skin microbiota has a fundamental and complex role in the control of skin immunity and beyond. Proper immune function of the skin is crucial, as its dysfunction is implicated in the pathogenesis of a variety of inflammatory skin disorders, including atopic dermatitis (AD). AD is a chronic inflammatory skin disease. Today, it is well established that patients with AD exhibit defects in the cutaneous barrier, in stabilizing skin integrity, and that the majority develops deviated innate and adaptive immune responses. As a result, increased susceptibility to cutaneous colonization and infection with bacteria is found in AD patients and the interactions between these microbes and the skin contribute to the development of chronic cutaneous inflammation [1]. Studies show that colonization with *Staphylococcus aureus* (*S. aureus*) correlates with AD severity and concentrations of proinflammatory cytokines such as IL-6 [2]. Following a disruption of the physical barriers a rapid, innate immune response needs to be initiated to prevent microbial invasion and replication. The recognition of pathogens is managed by the binding of substances derived of pathogens to so called pattern recognition receptors (PRRs) on immune cells. There are several classes of PRRs; among them Toll-like-receptors (TLR) and TLR2 has emerged as a principle receptor for Gram-positive bacteria, especially *S. aureus*, which is a potent provider of TLR2 ligands [3]. TLR2 has a unique ability to heterodimerize with TLR1 and TLR6. Recently we have identified that TLR2 ligands contribute to AD skin inflammation and promote transformation of Th2-dominated inflammation into its chronic form [4]. This occurs due to combination of the an early AD cytokine IL-4 and activation of TLR2 on skin immune cells causing an inhibition of anti-inflammatory IL-10, which is normally induced via TLR2, and as a consequence inflammation of AD is amplified and massively prolonged. Thus, overactivation of TLRs leads to the generation of strong pro-inflammatory signals with persistence of proinflammatory cytokines, such as TNFα and IL-6 and is associated with tissue damage. Therefore mechanisms to terminate and limit inflammation need to be effective. Indeed, in our work of Skabytska et al. [5], using a mouse model of cutaneous colonization [6], we have found that even limited cutaneous exposure to *S. aureus* induces severe inflammation. Consequently, in response to this strong inflammation, systemic immune suppression is established possibly as a regulatory feedback mechanism (Figure 1). Interestingly, TLR2/TLR6 but not TLR2/TLR1 ligands caused this immune suppression due to systemic induction of Gr1+CD11b+ myeloidderived suppressor cells (MDSCs) directly suppressing T cells. This result implies a heterogenic function of TLR2 heterodimers *in vivo*. TLR2/TLR6 recognize diacylated lipoproteins (Lpp), triacylated Lpp of bacteria bind TLR2/TLR1 heterodimers. So we propose that heterodimeric interaction of TLR2 with either TLR6 or TLR1 determine the outcome of the immune response. Furthermore, one can speculate that bacteria differ in their composition of TLR2/TLR6 versus TLR2/TLR1 ligands or that microorganisms can change their lipoprotein acylation patterns and consequently modulate immune responses. Indeed, it was shown recently, that the degree of Lppacylation depends on environmental factors and growth phase of the bacteria [7] and *in vitro* studies from different organs show a distinct ability in induction of gene expression, inflammatory mediators and Th responses of TLR2 heterodimer ligands. Thus, stimulation of TLR2 could have different immune consequences *in vivo*, including dampening of immune responses.

Interestingly, signals through TLR2 on skin cells, but not on hematopoietic cells, as well as cutaneous IL-6 induction were necessary and sufficient for the expansion of MDSCs and for MDSCs to exert their immune suppression in this context. Importantly, MDSCs are recruited to the skin inhibiting also anti-microbial immune responses, allowing bacteria and viruses to spread and to further aggravate cutaneous inflammation. These data from models are confirmed in human studies demonstrating MDSCs within peripherial blood mononuclear cells (PBMCs) and skin from AD patients, especially those with infectious complications such as eczema herpeticum. These patients suffer from spreading of herpes viruses due to immune suppression. As part of this pro-inflammatory cascade initiated by *S. aureus*-derived TLR2/6 ligands, IL-6 is upregulated within the skin, reaching significant levels also in peripheral blood, which is and responsible for MDSC accumulation. As part of the cutaneous inflammation, chemokines such as CCL22 and CCL28 are also induced in the skin contributing to the recruitment of MDSCs to the skin in the attempt to terminate or at least dampen inflammation. As this suppression is partly effective, infections can spread and superinfections develop in AD.
